# Correlation Between Anthropometric Measures and Biomarker Changes After Neoadjuvant Therapy With Tamoxifen or Anastrozole in Postmenopausal Women With Breast Cancer

**DOI:** 10.4021/wjon2010.06.224w

**Published:** 2010-05-19

**Authors:** Karine A. Cintra, Andre Mattar, Yong K. Joo, Alexandre Melitto, Ricardo Gonzales, Sueli Nonogaki, Fernando A. Soares, Angela F. Logullo, Luiz H. Gebrim

**Affiliations:** a Department of Gynecology, Federal University of Sao Paulo, UNIFESP, Rua Botucatu, 740, CEP 04023-900 Sao Paulo, SP, Brazil; b Department of Breast Medical Oncology, Perola Byington Hospital, Avenida Brigadeiro Luis Antonio, 683, CEP 01317-000 Sao Paulo, SP, Brazil; c Department of Pathology, AC Camargo Hospital, Rua Professor Antonio Prudente, 211, CEP 01509-010 Sao Paulo, SP, Brazil; d Deparment of Pathology, Federal University of Sao Paulo, UNIFESP, Rua Botucatu, 740, CEP 04023-062 Sao Paulo, SP, Brazil

**Keywords:** Breast cancer, Postmenopause, Tamoxifen, Anastrozole, Anthropometry

## Abstract

**Background:**

Epidemiological studies have reported positive associations between anthropometric measures and risk for developing breast cancers that express hormone receptors and associated mortality. However, the impact of nutritional status on the molecular response to endocrine therapy has yet to be described.

**Methods:**

Body mass index (BMI), waist circumference (WC), hip circumference (HP), and waist-to-hip ratio (WHR) were measured in patients with invasive ductal carcinoma (IDC) before and after neoadjuvant treatment with either tamoxifen or anastrozole, and a possible correlation with prognostic factors, as estrogen receptor (ER), progesterone receptor (PgR), and proliferative index (Ki-67), was analyzed. Fifty-seven patients with palpable ER-positive IDC were randomized into three neoadjuvant treatment groups and received anastrozole or placebo or tamoxifen for twenty-one days. Biomarker status was obtained by comparing the immunohistochemical evaluation of samples collected before and after treatment, using the Allred scoring system. Statistical analysis was performed using the Statistical Package for the Social Sciences (SPSS).

**Results and Conclusions:**

After treatment, the anastrozole group showed reduced ER and PgR expression (p < 0.05), and both the anastrozole and tamoxifen groups showed lower Ki-67 status. A significant reduction in PgR positivity (p < 0.05) was found in women with large WC and HC who were treated with anastrozole. Reduction in PgR positivity also tended to be associated with BMI (p = 0.09) in the anastrozole group. BMI, WC, HC and WHR correlated neither with biomarker levels in the tamoxifen and placebo groups nor with ER and Ki-67 status in the anastrozole group after primary endocrine treatment.

## Introduction

The incidence of breast cancer is still rising in developed countries, and the increasing prevalence of obesity may be an important contributing factor. Obesity has been shown to increase risk of breast cancer by 30 to 50% in postmenopausal women. Moreover, disease recurrence and mortality rates are also high in this population [1-4].

Several studies have investigated the relationship between anthropometric measures and breast cancer incidence. Colditz et al and Suzuki et al observed positive associations between body mass index (BMI) and risk for developing breast cancers that express hormone receptors, either estrogen receptor (ER) or progesterone receptor (PgR), in postmenopausal women [5, 6]. In agreement with these authors, Huang et al and Lahmann et al reported that postmenopausal women with large waist (WC) and hip circumferences (HC) had an increased risk of breast cancer [7, 8]. Borungain et al showed that the waist-to-hip ratio (WHR) was directly related to breast cancer mortality in this population [9].

Although adjuvant endocrine therapy has reduced breast cancer mortality, resistance to treatment is a challenge faced by physicians [10, 11]. Neoadjuvant endocrine therapy may downstage hormone-sensitive breast tumors, increasing the rates of breast-conserving surgeries or mastectomies in patients with tumors previously considered inoperable. Furthermore, preoperative endocrine therapy may be used to evaluate the tumor response to drugs at a molecular level over a short period of time, which may help in predicting the long-term outcome of the disease [12-14].

In the Immediate Preoperative Anastrozole, Tamoxifen, or Combined with Tamoxifen (IMPACT) trial, biological changes in biomarkers [ER, PgR, and proliferative index (Ki-67)] were compared after 2 and 12 weeks of treatment [15]. There was a significant suppression of Ki-67 in the anastrozole group compared to the tamoxifen or combination treatment groups. There was also an effective suppression of PgR in the anastrozole group [15]. Similarly, the Japanese Pre-operative Arimidex Compared to Tamoxifen (PROACT) trial showed a greater histopathological response rate and changes in ER and PgR status after 12 weeks of anastrozole treatment compared with tamoxifen treatment [16].

However, reports evaluating a possible relationship between anthropometric measures and endocrine response in breast cancer are scarce in the literature. Considering the epidemiological importance of obesity and breast cancer, the aim of this study was to increase knowledge about the relationship between measures commonly used to define obesity (BMI, WC, HC, WHR) and possible changes in breast cancer biomarker expression (ER, PgR and Ki-67) after three weeks of either anastrozole or tamoxifen neoadjuvant treatment in postmenopausal women with breast tumors expressing hormone receptors.

## Patients and Methods

### Patients

Eligible patients were postmenopausal women with untreated, invasive ER-positive (ER+) and/or PgR-positive (PgR+) operable breast cancer confirmed by incisional biopsy. Postmenopausal status was defined as patient ≥ 60 years old, or in the age range of 42 - 59 years and amenorrhea for ≥ 12 months with an intact uterus, or amenorrhea for < 12 months with postmenopausal levels of follicle-stimulating hormone (including patients who had undergone hysterectomy or bilateral oophorectomy).

### Study design

This was a prospective, randomized, double-blind, placebo-controlled trial in which 57 patients were randomly divided into 3 groups to receive a daily dose of anastrozole 1 mg (anastrozole group, *n* = 17) or placebo (control group, *n* = 25) or tamoxifen 20 mg (tamoxifen group, *n* = 15), for 21 consecutive days before surgery (mastectomy or breast-conserving). Baseline was defined as the first day of treatment.

Anthropometric data were obtained on hospital admission according to the World Health Organization guidelines [17] as follows: weight was measured in kilograms (kg) to the nearest 0.1 kg using a Filizola® scale; height was measured in centimeters (cm) with a stadiometer coupled to the scale; and waist and hip circumferences were measured in centimeters with a flexible, non-stretch tape. BMI was calculated by the formula BMI = weight/(height)^2^, and waist-to-hip ratio was the ratio between the waist circumference and hip circumference.

The ER, PgR and Ki-67 status were determined by immunohistochemistry in the laboratory of pathology of the Federal University of Sao Paulo (UNIFESP) and A. C. Camargo Hospital, using the primary antibodies SP1 (NeoMarkers cat# RM-9101-S), PgR 636 (Dako cat# M3569), and MIB-1 (Dako cat# M7240). Tissue samples were obtained at baseline and after three weeks of treatment.

This study was approved by the Research Ethics Committee of the Perola Byington Hospital and Federal University of Sao Paulo (UNIFESP), Brazil, (process number 0904/04) functioning according to the 3rd edition of the Guidelines on the Practice of Ethical Committees in Medical Research issued by the Royal College of Physicians of London. Written informed consent was obtained from each patient after full explanation of the purpose and nature of all procedures used. Patient anonymity was assured.

### Statistical analysis

Data were recorded on Excel® spreadsheets and statistical analysis was performed using the Statistical Package for the Social Sciences (SPSS) for Windows. Statistical significance was set at p < 0.05 and confidence intervals (CI) were set at 95%. Frequency tables were produced to describe the study population. Comparison of biomarker status (ER, PR and Ki-67) before and after treatment was performed using analysis of variance (ANOVA). The Kruskal-Wallis and Mann-Whitney tests were used to investigate the association between anthropometric measurements (BMI, WC, HC and WHR) and biomarker changes (ER, PgR and Ki-67) within groups before and after treatment.

## Results

Table 1 summarizes the clinical characteristics of the study population. Mean age at breast cancer diagnosis was 66 years, and mean age at menopause was 48 years. The mean values of anthropometric measurements were: body weight of 69.3 kg, height of 1.56 m, BMI of 28.2 kg/m^2^, WC of 97.1 cm, HC of 100.9 cm, and WHR of 0.96.

**Table 1:** Clinical Characteristics of the Study Population

Variable	N	Mean	SD	Minimum	Median	Maximum
Age (years)	57	66.25	9.95	42	67	87
Tumor size (cm)	57	4.00	1.06	2.5	4	8
Menarche (years)	56	13.00	1.68	9	13	16
Menopause (years)	56	48.04	5.43	39	50	60
Parity (number of children)	57	3.33	3.29	0	3	13
Height (cm)	57	156.75	6.39	139	156	169
Weight (kg)	57	69.26	15.82	40	69.5	120
BMI (kg/m^2^)	57	28.18	6.23	16.6	28.6	53.3
HC (cm)	57	101.22	10.90	79	102	128
WC (cm)	57	97.11	15.48	68	97	160
WHR	57	0.96	0.09	0.8	1.0	1.4

N: number of participants; SD: standard deviation; BMI: body mass index; HC: hip circumference; WC: waist circumference; WFR: waist-to-hip ratio.

The mean tumor size was 4 cm, and 76% of the tumors were classified as nuclear grade 2 invasive ductal carcinomas (IDCs). The majority of patients had stage II (stage IIA, 60%; stage IIB, 25%) tumors with ER (84%) and PgR (59%) expression.

**Figure 1:**
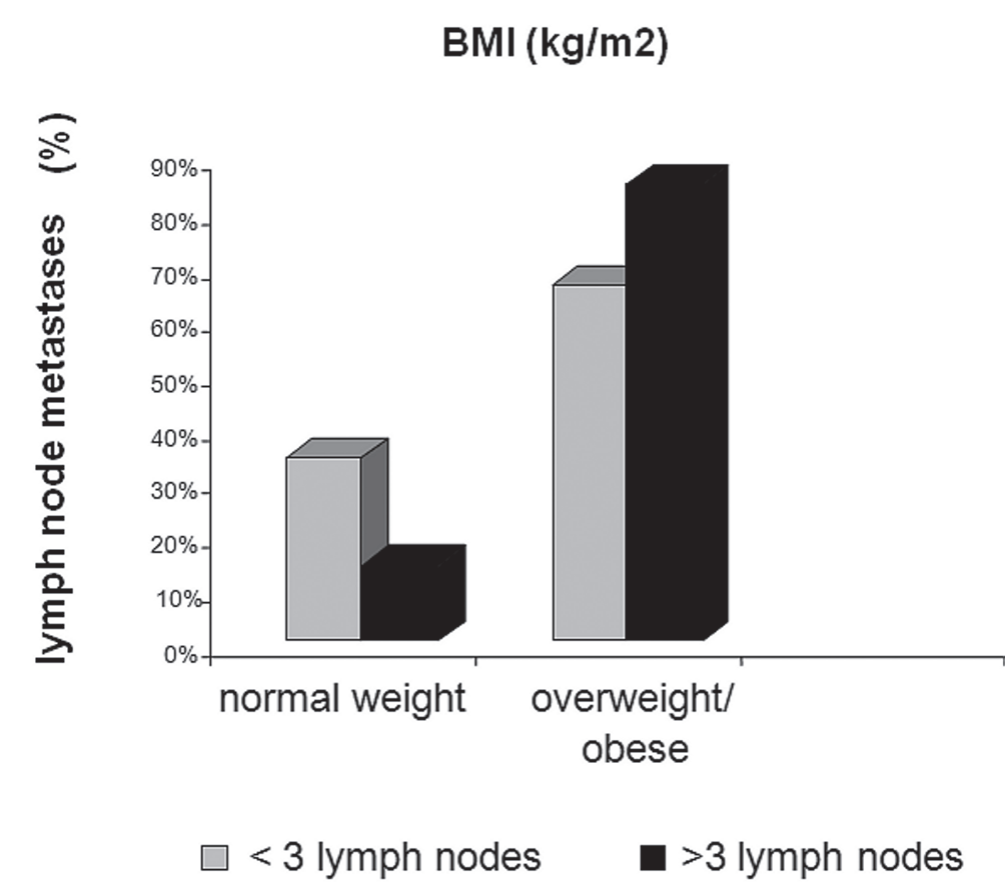
Body mass index (BMI, kg/m^2^) versus Lymph node metastases (%).

Results revealed that anthropometric measures (BMI, WC, HC, and WHR) did not correlate with clinical stages of breast cancer. Nevertheless, BMI was significantly associated with axillary lymph node status (Fig. 1). It was observed that 64% of the patients who had more than three lymph nodes involved were overweight (BMI ≥ 25 kg/m^2^), while only 22% of the patients with less than three metastatic lymph nodes were overweight (p = 0.039).

**Figure 2:**
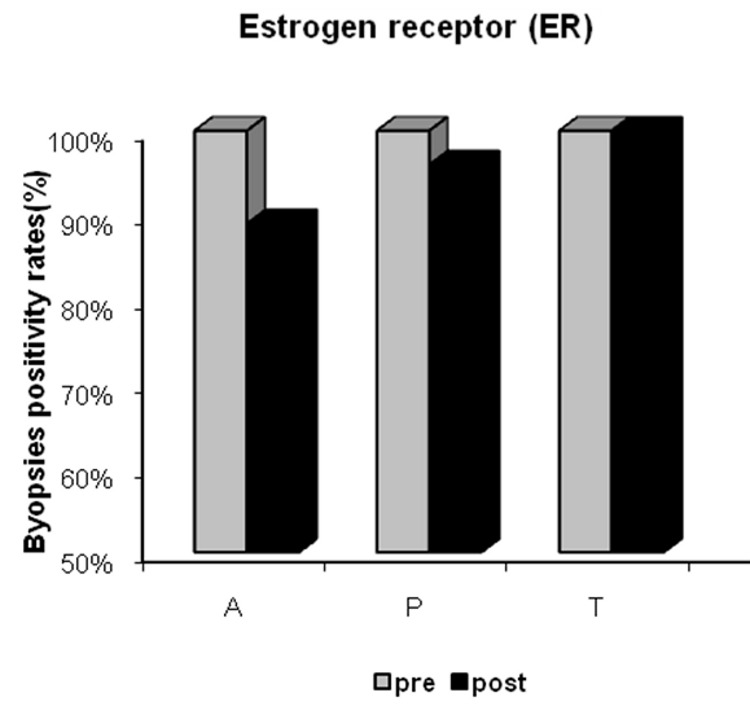
Estrogen receptor (ER) positivity in the anastrozole (A), placebo (P) and tamoxifen (T) groups pre- and post-treatment.

Rates of ER positivity were 100%, 96% and 100% at baseline, and 89%, 96% and 100% after treatment in the anastrozole, placebo, and tamoxifen groups, respectively. Despite the slight decrease in ER positivity in patients treated with anastrozole for 21 days, no statistically significant difference was found between pre- and post-treatment rates (Fig. 2).

**Figure 3:**
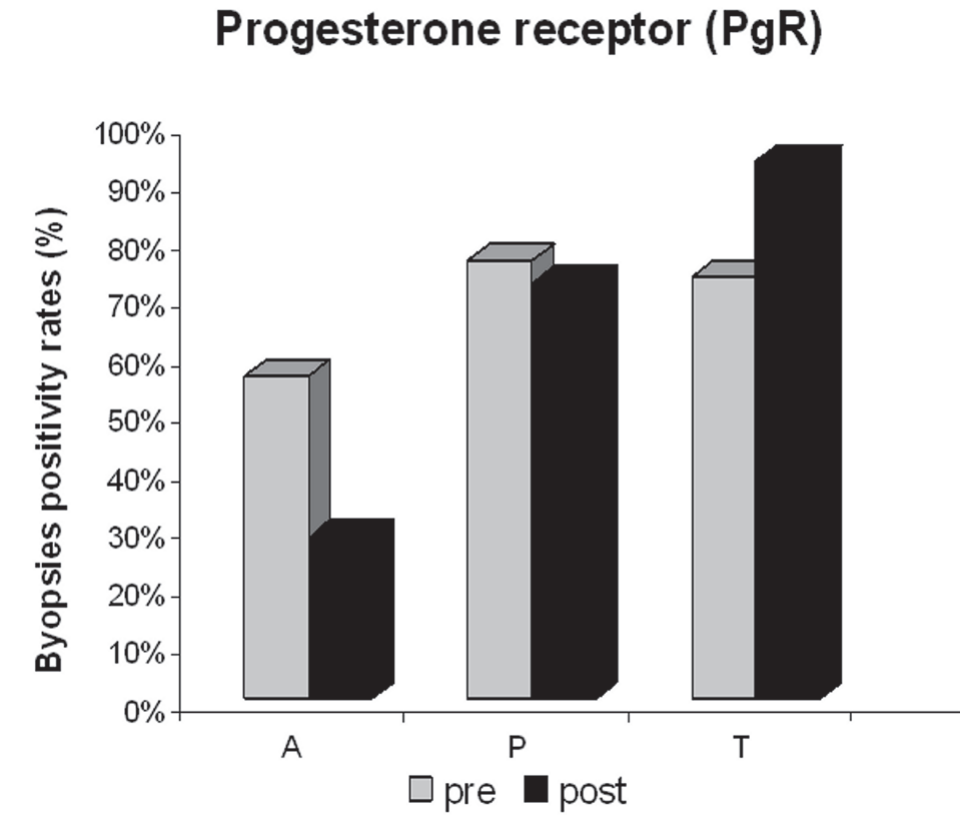
Progesterone receptor (PgR) positivity in the anastrozole (A), placebo (P) and tamoxifen (T) groups pre- and post-treatment.

**Figure 4:**
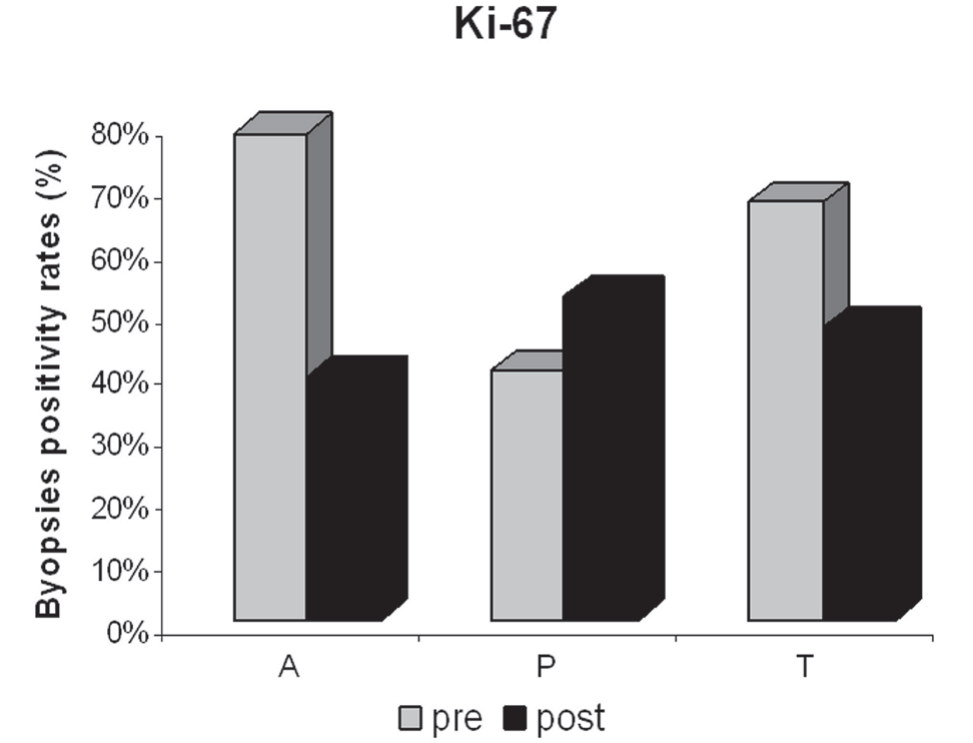
Proliferative index (Ki-67) positivity in the anastrozole (A), placebo (P) and tamoxifen (T) groups pre- and post-treatment.

**Figure 5:**
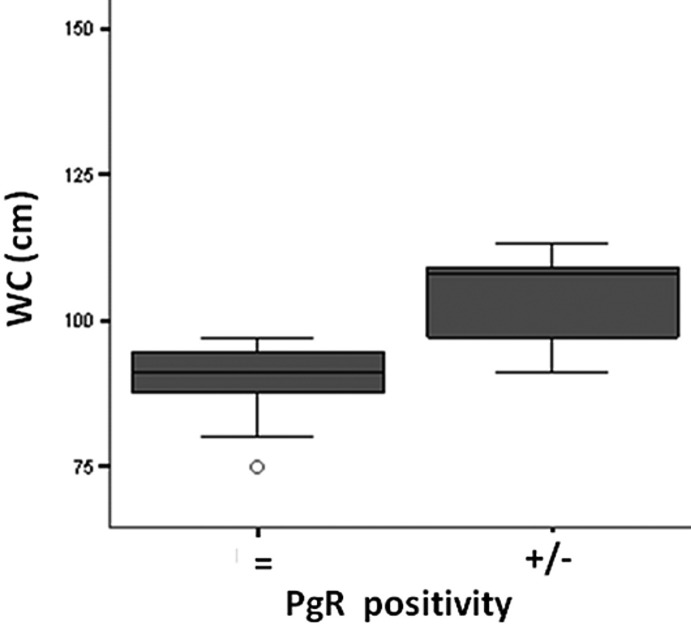
Waist circumference (WC, cm ) versus Progesterone receptor (PgR) positivity after anastrozole treatment.

**Figure 6:**
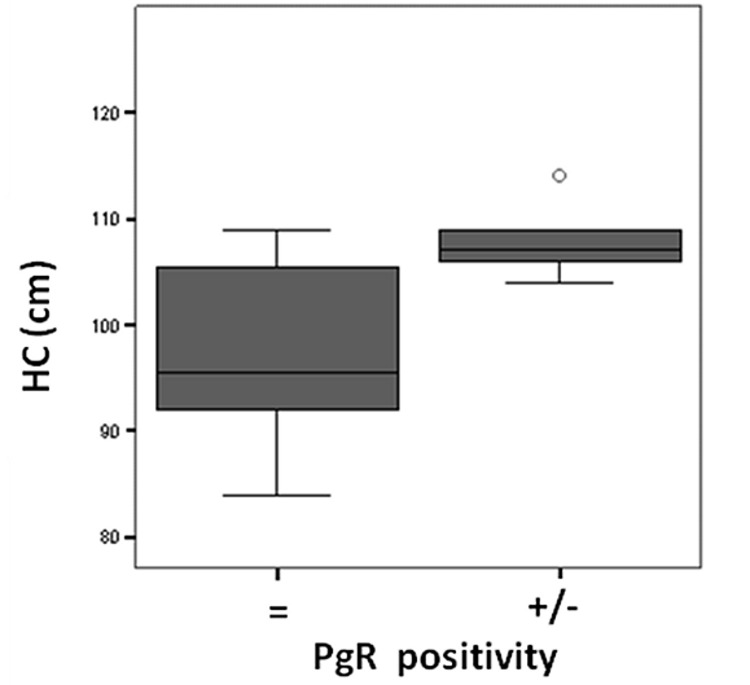
Hip circunference (HC, cm) versus Progesterone receptor (PgR) positivity after anastrozole treatment.

There was a significant post-treatment reduction in PgR (Fig. 3) and Ki-67 positivity (Fig. 4) in the anastrozole group compared with the other groups. Rates of PgR positivity were 56%, 76% and 73% at baseline, and 28%, 72% and 93% after treatment in the anastrozole, placebo and tamoxifen groups, respectively. There was a significant post-treatment reduction in PgR positivity in women with large WC and HC (p < 0.05), as shown in Figure 5 and Figure 6. BMI also tended to be associated with this biological response (p = 0.09).

At baseline, Ki-67 was positive in 78%, 40% and 67% of the samples from the anastrozole, placebo and tamoxifen groups, respectively, and after treatment, in 39%, 52% and 47% of the samples, respectively. Post-treatment reduction in Ki-67 positivity was not associated with anthropometric measures in the tamoxifen and anastrozole groups.

There was a positive association between WHR and PgR. Women with high WHR had high rates of PgR positivity, although WHR was not significantly associated with biomarkers after neoadjuvant endocrine therapy with either tamoxifen or anastrozole (p = 0.39).

## Discussion

The anastrozole treatment led to a significantly greater reduction in Ki-67 and PgR positivity than the tamoxifen and placebo treatments, which is in agreement with previous studies [15, 16]. Our results showed that patients with large WC and HC, and probably those with high BMI (p = 0.09) had a better PgR response to anastrozole treatment. Changes in ER and Ki-67 were not significantly associated with anthropometric measures.

Despite recent advances in molecular biology and genetics of breast cancer, no validated surrogate markers for predicting long-term prognosis have been identified [18].

Approximately 75% of primary breast cancers express ER, and more than half of these cancers also express PgR [19, 20]. Both the ER and PgR are prognostic factors, and ER status is a strong predictor of response to endocrine therapy. The predictive power of PgR was recently questioned due to differences in results obtained from large clinical trials [21-23]. Results from the Arimidex, Tamoxifen Alone, or in Combination (ATAC) trial showed that depending on ER/PgR status, anastrozole had a modest advantage over tamoxifen in the ER+/PgR+ group, while anastrozole provided a major benefit to the ER+/PgR- group [24]. Nevertheless, in the Breast International Group (BIG) 1-98 trial, the degree of benefit accrued from letrozole compared to tamoxifen did not change based on the PgR status [25]. Moreover, in the sequential MA-17 trial, the ER+/PgR+ group derived more benefits from letrozole than the ER+/PgR- group did from tamoxifen [26]. Therefore, both the ER and PgR expressions are still important markers for clinical decision-making in breast cancer.

Some studies have shown that ER and PgR status can change with the natural history of the disease or during treatment. In general, reductions in ER levels are small, and complete ER loss is uncommon. In contrast, a reduction in PgR levels after tamoxifen therapy has been associated with tamoxifen resistance, with complete loss of PgR expression when resistance develops [27, 28]. Similarly, we observed a slight reduction in ER positivity after neoadjuvant anastrozole treatment, but no changes were seen after either tamoxifen or placebo treatment. However, there was a significant reduction in PgR positivity after neoadjuvant anastrozole therapy and increased positivity after tamoxifen treatment, which is consistent with the findings of the IMPACT trial after two weeks of treatment [15]. The mechanism for the increase in PgR in patients treated with tamoxifen for such a short period is unclear, but a hypothesis of an agonistic effect at the beginning of treatment has been accepted [20].

Although several studies have demonstrated a positive association between anthropometric measures and ER/PgR expression in postmenopausal breast cancer [29-34], no studies on the correlation between these indirect measures of body fat distribution and molecular response to endocrine therapy were found in the literature.

Obesity in postmenopausal women has been associated with increased exposure to estrogen, insulin, and insulin-like growth factors (IGF) that are associated with breast carcinogenesis [35, 36]. Furthermore, some studies have shown a consistent association between obesity and lymph node status in patients with ER-positive tumors [37]. In agreement, the present study showed a higher number of metastatic lymph nodes in overweight and obese patients than in normal weight patients. Moreover, it is well known that PgR is an estrogen-regulated protein, whose expression indicates a functional ER pathway [23]. Therefore, the marked reduction in PgR positivity in patients with large WC and HC in the anastrozole group could be the result of a better peripheral action of the aromatase inhibitor in the adipose tissue. The lack of correlation between ER and anthropometric measures might be related to the short period of treatment.

Moreover, Ki-67 has been widely studied and used as both a prognostic marker and as a biological marker to compare drug effects. More recently, results from the IMPACT trial showed that the absolute level of Ki-67 after 2 weeks of endocrine therapy is an independent predictor of disease-free survival [38, 39]. These results also showed that a reduction in Ki-67 levels did not correlate with anthropometric measures in all the treated groups, which could reinforce its importance as an independent prognostic marker.

The association between clinical parameters and molecular response to treatment of breast cancer may be important in the decision-making process regarding adjuvant therapy. In the present study, results suggest a superiority of anastrozole over tamoxifen in postmenopausal breast cancer patients with large WC and HC. Considering the relevance of breast cancer biomarkers and current epidemiological importance of obesity, further studies are needed to evaluate the usefulness of anthropometric measures in clinical decisions.
